# Feasibility assessment of textile electromyography sensors for a wearable telehealth biofeedback system

**DOI:** 10.1017/wtc.2025.10012

**Published:** 2025-06-16

**Authors:** Beomjun Ju, Jasper I. Mark, Seonyoung Youn, Prateeti Ugale, Busra Sennik, Brady Adcock, Amanda C. Mills

**Affiliations:** 1 Department of Textile, Engineering Chemistry and Sciences, Wilson College of Textiles, North Carolina State University, Raleigh, North Carolina, USA; 2 Impulse Wellness, Chapel Hill, North Carolina, USA

**Keywords:** biosignals, electromyography, e-textiles, inkjet printing, screen printing, telehealth system

## Abstract

Our study investigated the efficacy and feasibility of screen-printed and ink-printed textile-based dry electrodes for electromyography (EMG) acquisition, marking a novel step in wearable telehealth (TH) system integration. We controlled the design and fabrication conditions of these textile EMG sensors, including electrode area and sizing, ensuring optimal contact pressure. Skin-electrode impedance for all designs was evaluated, and a 20 mm electrode diameter was deemed material-efficient and design-effective. When compared with standard 20 mm wet electrodes, our EMG sensors with the screen and inkjet-printed dry electrodes exhibited comparable signal-to-noise ratios (SNR_dB_) to the conventional wet electrode (26 dB) with a peak of 25 dB, and 23 dB, respectively, emphasizing their reliability. Our research identified a 10% optimal strain by sizing for EMG performance across both printing techniques. These revelations support the future design of dependable, reusable dry textile electrodes, addressing challenges faced by wet electrodes. Additionally, the developed dry electrodes, when equipped with a Bluetooth-enabled amplifier puck mitigate common EMG challenges such as motion artifacts while promoting user comfort, which leads to an elevated user experience during EMG biosignal collection. The integration of the developed garment-based electrodes with available commercial technologies holds promise for enhancing TH systems and user engagement in wearable health monitoring.

## Graphical abstract

**Figure:**
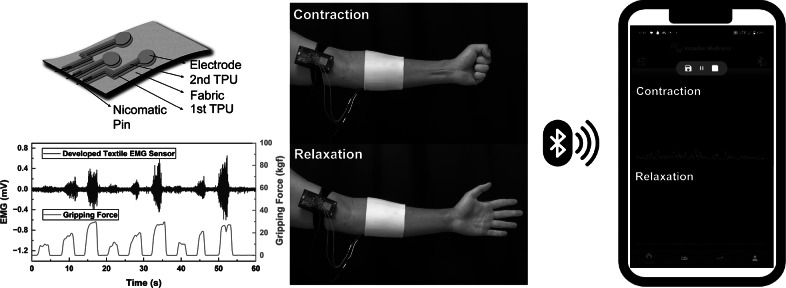

## 1. Introduction

The exponential growth of wearable technologies in recent years has reshaped the landscape of healthcare, transitioning from mere activity tracking to sophisticated medical applications. New medical-grade wearables are transforming disease diagnostics, monitoring, and chronic treatment strategies by fusing with common consumer wearables (Jin et al., 2017). Since the initial development of wearable technologies that are most widely in the form of smartwatches, there has been significant effort towards the development of garment-based wearables for more seamless daily integration (Lam et al., 2022). Simultaneously, the demand for telehealth (TH) tools has surged as technology-enabled solutions promote remote delivery of diagnostic and treatment services, offering personalized, convenient, and engaging care in the comfort of one’s own home (Ding et al., 2021).

For example, stroke, which is a leading cause of disability worldwide (Virani et al., 2021), profoundly impacts patients’ motor functions and often necessitates prolonged rehabilitation to facilitate optimal recovery. Rehabilitation efforts are often hindered by socioeconomic, geographical, and psychological barriers, further limiting access to care and driving patient non-compliance with treatment following hospitalization discharge. One potential solution to these challenges is the integration of TH technologies with garment-based sensors for biofeedback telerehabilitation training (Oliver et al., 2018). These wearable sensors can gather a myriad of topical biosignals such as heart health information derived from electrocardiographic (ECG) signals and notably muscle activation via electromyography (EMG) signals (Acar et al., 2019). TH systems incorporating biosignal acquisition, particularly EMG for individuals with neuromuscular disorders, can serve as instrumental tools for quantifying and developing effective neuro-rehabilitative treatments. Such technologies incorporating muscle activity visualization and analysis may provide key insights into the underlying mechanisms resulting in motor deficit following neural injury and subsequent targets for treatment (Hughes et al., 2014). Additionally, such technologies may become particularly indispensable when paired with biofeedback, empowering users by visualizing even the minutest muscle activations, thus acting as a motivational driver and providing quantifiable metrics on a patient’s motor function. Despite modern advances in EMG technology, the establishment of guidelines for EMG implementation through the European recommendations for Surface EMG Non-Invasive Assessment of Muscles (SENIAM), and a significant body of literature supporting EMG use, its adoption in clinical practice remains limited (Stegeman and Hermens, 2007).

Traditional EMG technologies employ adhesive-based single-use wet electrodes. While effective in controlled settings, these sensors pose numerous challenges. They may impede movement, detach from the skin, or require intricate wire management to prevent motion artifacts. Furthermore, recording consistent muscle activity over different sessions is challenging, leading to inconsistencies in longitudinal EMG data. There have been approaches to developing dry electrodes based on pure Ag (Jiang et al., 2020), Ag/AgCl paste (Li et al., 2021), liquid metals (Li et al., 2022), graphene (Ozturk et al., 2021), PEDOT:PSS (Pani et al., 2019), and conductive thread (Lyons et al., 2023) to defy the challenges posed by conventional single-use wet electrodes. In this work exploring the development of comfortable and reliable biosensors, electronic textiles (e-textiles) based on printing conductive inks are promising techniques for fabricating scalable dry electrodes that circumvent the aforementioned limitations. Textile-based printed dry electrodes emerge as an affordable and enduring platform for wearable health monitoring due to ease of placement and re-usability. (Yokus and Jur, 2016) Moreover, they can be tailor-made and arrayed to cover entire extremities, facilitating extensive motor group visualization and thus presenting a potential to revolutionize EMG measurements in diverse settings across rehabilitation clinics, research labs, and home-use scenarios.

The journey of embedding electronics into textiles is not without hurdles. Garments based on e-textiles, especially biopotential sensing devices, are challenging to prototype, as researchers must intricately balance nuances between performance, comfortability, and data quality (Li et al., 2021). Chiefly, issues with high skin-electrode impedance and motion artifacts have been a concern. The cornerstone for successful e-textile garments lies in achieving optimal contact pressure (CP) between the sensor and skin, especially during motion. Prevailing research underscores the significance of adequate CPs, suggesting that optimal ranges of 0.5–3 kPa (Kim et al., 2020; Taji et al., 2018; Takeshita et al., 2019) are required to procure high-quality biopotentials’ waveforms devoid of motion artifacts.

The novelty of this research lies in the development of a wearable EMG measuring system. Our approach is distinct in several keyways. First, we utilized advanced printing technologies to fabricate dry electrodes, which are integrated with textiles, allowing for flexible and customizable electrodes. This is significantly different from traditional methods. Our research explores various form factors for these printed electrodes, focusing on their integration with textiles, an innovation not extensively explored in the literature. Recently, screen printing and inkjet printing methods have been explored for textile-based EMG sensors demonstrating a textile interface for high-density EMG recordings using screen printing (Murciego et al., 2023), whereas Kim et al.’s research presented textile-based dry electrodes with comparative surface EMG analysis (Kim et al., 2022). Similarly, conductive inks utilizing graphene nanoplatelets (Marra et al., 2021), graphene-silver composites (Karim et al., 2019), and reactive inkjet printing techniques for silver electrical circuits (Xiao et al., 2022) have been reported. While these studies provided significant groundwork, our work is distinctive by specifically investigating the effects of electrode sizes and textile strains on EMG signal quality, thus optimizing electrode design and performance for enhanced biosignal fidelity and wearability. Secondly, we specifically investigate the effects of different electrode and textile sizes on signal quality, optimizing the design for better performance. By combining these elements, our work contributes a novel approach to the state of the art in wearable EMG systems, offering improved flexibility, customization, and signal quality through the use of printed flexible electrodes on textiles.

In this paper, we proposed the development of a textile EMG sensor system based on printed dry electrodes for TH application. We explored the effect of electrode dimension and armband tightness on the signal-to-noise ratio (SNR_dB_) of collected EMG to define optimal system design specifications. Additionally, we integrated the textile EMG armband with the previously developed Bluetooth Electromyography-based Stroke Therapeutic (BEST) system (Impulse Wellness, Raleigh, NC). Then, we validated its performance as a wearable muscle-sensing device and user-friendly mobile biofeedback application used to improve the daily living of neuro-compromised individuals.

## 2. Materials and methods

### 2.1. Textile EMG sensor fabrication

**Figure 1.**
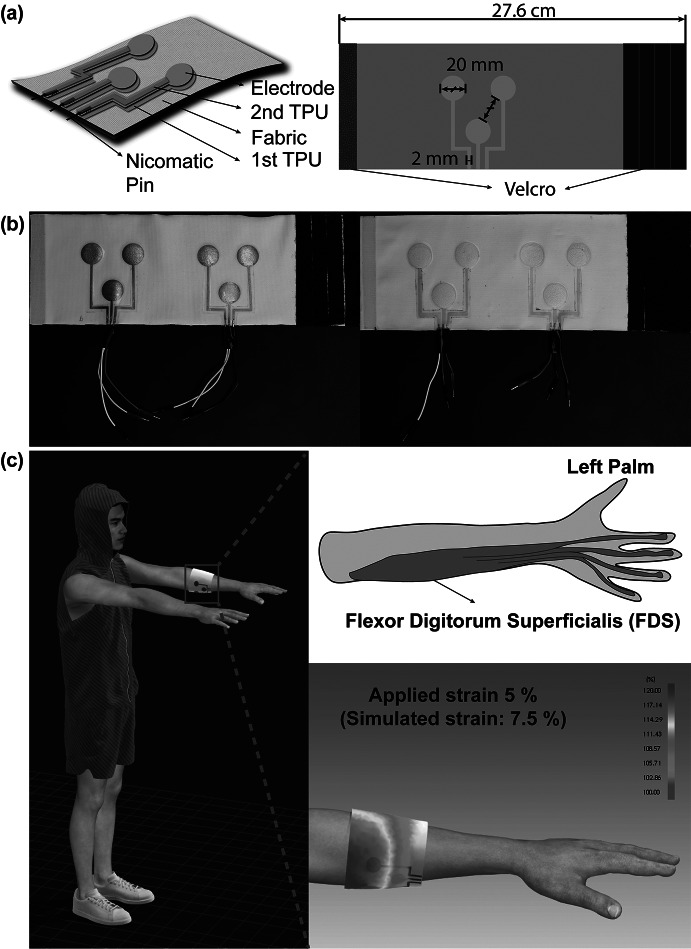
(a) Schematic illustration of the fabrication process and final structure of the textile EMG sensor. (b) Optical images of the textile EMG sensors with inkjet-printed dry electrodes (left) and screen-printed electrodes (right). (c) 3D-simulated illustration and strain map by CLO3D showcasing the placement of textile EMG sensors at flexor digitorum superficialis as a target muscle.

For screen-printed electrodes, Ag/AgCl ink (Dupont 5880) was applied onto a thermoplastic polyurethane (TPU) substrate (Dupont Intexar™ TE-11C) via a 200-count steel mesh, following the method outlined in Yokus et al. (2016). The inkjet-printed electrodes were fabricated using metal–organic deposition Ag ink (Liquid X Printed Metal) as shown in Figure 1. Prior to printing, the ink was passed through a 0.1 μm PTFE filter. A Fujifilm Dimatix (DMP-2850) inkjet printer was then employed to deposit the filtered ink onto the TPU substrate. Utilizing a cartridge head equipped with 12 nozzles, drop generation was achieved by modulating the voltage of the piezoelectric actuators to 26.5 V at a frequency of 23 kHz. Each droplet had a jetting duration of 7 μs and was dispensed with a resolution of 1693 dpi, maintaining a spacing of 15 μm between drops. Throughout the process, a consistent 2 mm distance was maintained between the substrate and the cartridge head. After printing 30 paths on the substrates, the inkjet-printed paths were annealed in a vacuum oven at 130 °C for 15 min, ensuring the completion of the chemical reaction and optimizing electrical properties (Kim et al., 2021; Ju, 2023).

Three electrodes designed for bipolar configuration and grounding constitute a set that is then strategically placed on an armband. We explored the effects of varying electrode diameters on signal quality by designing electrodes with 10, 20, and 30 mm diameters. An inter-electrode distance of 20 mm was maintained to ensure compliance with SENIAM project recommendations. These electrode designs were created by Adobe Illustrator. A laser cutter (Epilog Mini 18, 40 Wt) was used post-printing to trim the TPU-based electrodes, keeping a 2 mm offset from the conductive trace’s edge. For external connectivity, metal crimping pins (Nicomatic CRIMPFLEX™) were affixed to the interconnect ends.

**Figure 2.**
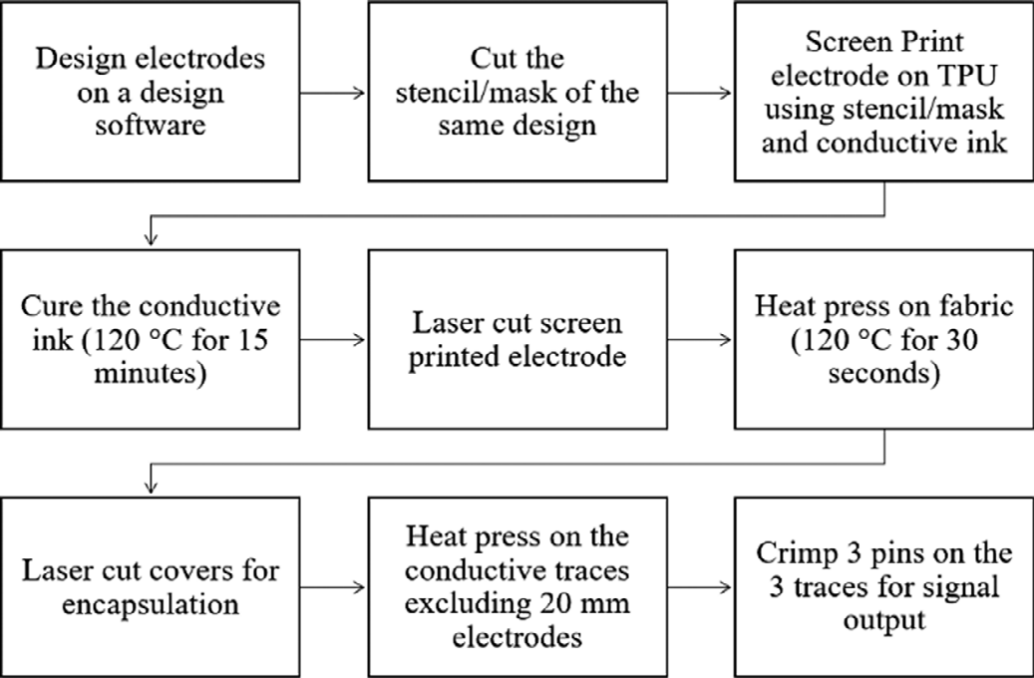
Block diagram of screen-printed EMG sensor fabrication process. The process for fabricating an ink-jet printed EMG sensor is identical except for the printing process.

The employed textile armband was crafted from a single jersey knit textile comprising 85% polyester and 15% spandex. It has an area density of 240 g/m^2^ and a thickness of 0.48 mm. The fabric was cut into rectangular pieces and sewn together, aligning the seam with the wale (warp) direction. Meanwhile, the course (weft) direction wrapped around the arm’s circumference. The resultant armband was tailored to the wearer’s forearm, measuring 27.6 cm for the subject. Additionally, to explore the textile’s design impact on sensor performance, Velcro strips were attached at the ends of the armbands, enabling manual strain adjustments ranging from 5 to 20% of the armband circumference. To showcase the placement of textile EMG sensors at a target muscle with strain map while wearing it, simulations were created using CLO3D (CLO Virtual Fashion). The printed electrode patterns were secured to the textile armband by heat pressing for 2 min at 130 °C. An extra TPU layer was then heat-laminated atop the printed patterns for 2 min at the same temperature, ensuring the conductive interconnects were encapsulated for protection. Notably, the sensing region of the Ag/AgCl electrode remained exposed for optimal EMG signal capture from the body. The comprehensive process is diagramed in Figure 2.

### 2.2. EMG measurement protocol

For the EMG assessment, we involved one male subject aged between 30 and 35 years with no known health issues. Ethical Committee approval was obtained with the IRB number 16707. The effect of electrode area and armband design and efficacy of the dry electrodes on electromyography (EMG) signal was investigated utilizing a widely used biosignal acquisition tool (MP35, BIOPAC). EMG was collected at a sample rate of 2000 Hz with a notch at 60 Hz. The garment-sensor system was applied to the participant’s forearm, whereas the position of the line between the two bipolar electrodes parallel to the direction of the muscle fibers of the flexor digitorum superficialis (FDS) of the left arm, as defined by SENIAM project recommendations (Stegeman and Hermens, 2007). For gripping motions, the FDS is a key extrinsic hand muscle responsible for flexing the joints of the primary fingers (Lung and Burns, 2022). Our primary aim was to quantitatively evaluate these hand motions by capturing EMG signals from the FDS. The subject was seated on a chair with the elbow flexed at a slightly obtuse angle. Upon donning the electrode sleeve, the participant engaged in a 5-min stabilizing period to ensure consistent contact between the electrode-skin interface during EMG measurement. Afterward, the participant performed three sets of 25, 50, and 75% maximum voluntary contraction (MVC) isometric hand squeezes with visual force output feedback provided. Contractions were interleaved with three-second periods of rest. MVC was determined by the use of a digital dynamometer. The above assessment was repeated with the varying-sized electrodes and strains.

### 2.3. Contact pressure and skin impedance measurement

Contact pressure was measured across each session with various conditions by use of a Kikuhime pressure sensor (TT MediTrade) with a 35 mm diameter placed between the skin and the armband. The pressure sensor was located at the center of the electrode configuration. The measurements were repeated three times to ensure consistent data, and the acquired values in mmHg were converted to kilopascals for a more standardized comparison. The impedance of the skin–electrode interface was assessed by the use of a potentiostat (Gamry Reference 600). Potentiostatic EIS experiment under the Electrochemical Impedance category was used for evaluation. This assessment was carried out on a male subject’s left arm using the same three-electrode configuration as deployed in measuring EMG signals, wherein the counter and working electrodes carry current, and the working and reference electrodes measure voltage.

### 2.4. Signal-to-noise (SNR) calculation

SNR_dB_ was calculated for the maximal 75% MVC target isometric contraction. This involved computing the root mean square (RMS) of the contraction and resting periods as seen in Equation 1. Data were visually inspected to ensure no muscle activation or force output was present during the rest sample. Specifically, the contraction period was selected as the window in which force data was within 5% of the target 75% MVC over 2500 ms, ensuring enough time for muscle ramp up during the motor recruitment period. As a control analysis, the EMG signals within these periods were confirmed to be greater than 2.5 standard deviations above the baseline noise, ensuring an active myoelectric segment. The rest segments were defined as the first 500 ms to 3000 ms of the recording session to account for any edge effects. The participant was instructed to remain at rest during the first 3 s of the recording, and this period was confirmed by no force output on the dynamometer. The EMG data was normalized to the maximal value for each recording and notch filtered at 60 Hz prior to computing SNR_dB_.(1)

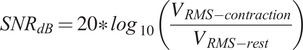



### 2.5. PCB design for amplifier puck

The embedded BEST system consists of a PCB containing a battery and various microchips used for signal amplification, acquisition, and wireless transmission. The purpose of this hardware includes (1) collecting EMG signal, (2) amplifying and filtering the EMG data, (3) converting EMG electrode signals to digital values, and (4) transmitting this preprocessed data to an external Bluetooth terminal (the mobile application) as demonstrated in a later section. A power switch controlling a buck-boost converter supplied a consistent 5 V source to the PCB. The amplifier chip takes raw EMG waveforms ranging from 0.1 mV to 10 mV and increases the amplitudes to a range of 1 V to 5 V. The output of this filter leads to an Analogue to Digital Converter (ADC) chip, which converts the signals to Inter-Integrated Circuit (I2C) format and hands them to the BLE-capable embedded processor for transmission. This processor is capable of entering BLE pairing mode via a button. The PCB was designed in Fusion360 (AutoDesk, San Francisco, CA).

### 2.6. Signal process for mobile application

Collected EMG signals were preprocessed with a 60 Hz notch filter and bandpass filter between 10 and 250 Hz. Additionally, the waveform was passed through a Hilbert transform, rectified, and smoothed along a 100 ms moving window. The Hilbert transform was applied to better compute the signal envelope and to ensure smoothing would not decimate the data. These steps were performed to ensure efficient data display within a mobile compute device with low latency.

## 3. Results and discussion

### 3.1. Textile EMG sensor design and fabrication

To evaluate the effectiveness of the printed dry electrodes, textile EMG sensors were produced using dry electrodes of varying diameters: 10 mm, 20 mm, and 30 mm. These diameters were chosen based on the SENIAM recommendations (10 mm), conventional wet electrode dimensions (20 mm), and the recommended diameter as previously demonstrated by other researchers (30 mm) (Li et al., 2022) to find the optimum dimension for our wearable TH biofeedback system as depicted in Table 1. Electrodes were labeled based on their printing method and diameter; for example, S10 refers to screen-printed electrodes with a 10 mm diameter, while I20 refers to inkjet-printed electrodes with a 20 mm diameter.

**Table 1.** Fabrication conditions of textile EMG sensors

Fabrication conditions		Applied strain (%)			
		5	10	15	20
**Electrode diameters (mm)**	**10**	S/I10–5	S/I10–10	S/I10–15	S/I10–20
	**20**	S/I20–5	S/I20–10	S/I20–15	S/I20–20
	**30**	S/I30–5	S/I30–10	S/I30–15	S/I30–20

*Note:* The textile EMG sensors were fabricated with the various diameters of the printed electrodes, applied strain, and printing techniques based on factorial experimental design. (S: Screen-printed electrodes, I: Inkjet-printed electrodes).

A comparative analysis was conducted on the EMG sensing performance of screen-printed versus inkjet-printed electrodes. Screen-printed electrodes demonstrated dependable conductivity on textiles but were more prone to deformation due to their relative thickness, approximately 20 μm (Yokus et al., 2016). In contrast, inkjet printing, with a layer thickness from 250 nm to 2.5 μm (Kim et al., 2019), allows for thinner, more conformal layers on substrate surfaces, attributed to the use of less viscous inks.

Furthermore, the structure of interconnects and the integration method for external connection to the amplifier puck were integral considerations. Given that textiles can undergo about 20% strain during various human activities and human form factors, electronics embedded within might experience up to∼20% strain (Mattmann et al., 2007). Our approach, illustrated in Figure. 1 (a), involves a multilayered structure: printed electrodes on TPU, further heat-laminated layer using additional TPU. This structure ensures the interconnects are stretchable, supporting functionality while also allowing freedom of movement for wearer comfort. We chose a textile substrate because fabrics can be tailored to cover broader muscle areas, enhancing the granularity of motor group visualizations. We opted for single jersey knit fabric because the looped knit structure provides inherent elasticity, making it suitable for textile EMG sensors that need compression for signal capture. To ensure optimal contact pressure for high-quality biopotential signals, previous methods sewed fabric armbands of varying sizes. This method, however, is both non-adjustable and time-intensive. Our solution, depicted in Figure 1 (b) and (c), was to affix Velcro strips at the armband ends, providing users with an adjustable and more comfortable wearing experience.

We designed the interconnects to extend from the electrodes to the armband’s length edge, promoting easy external connections and reducing motion-related disturbances. While many researchers have employed metal snaps to establish these connections, these are bulkier and can compromise the interconnect due to their expansive contact area during mechanical crimping (Stanley et al., 2022). Our use of Nicomatic pins mitigates such damage due to their reduced size and contact area. Despite being crimped, these smaller pins reduce the overall pattern size and enhance durability against deformation.

### 3.2. Signal quality of textile EMG sensor

**Figure 3.**
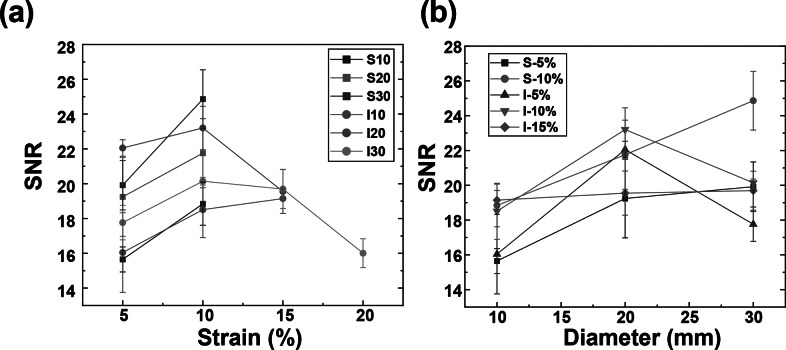
(a) SNR_dB_ of raw EMG signals from textile EMG sensor with different strain levels, and (b) Average contact pressure between skin and electrodes according to the strain level controlled by the velcro strips on the armband.

Our primary aim was to quantitatively evaluate these hand motions by capturing EMG signals from the FDS. We measured the FDS EMG signal during the target grip task with the visual force output provided, as shown in Figure 3(a). During contraction, the muscle fibers of the FDS are activated, resulting in measurable EMG activity. As the magnitude of gripping force increases, the amplitude of the EMG signal also increases, whereas this relationship is largely linear up to a saturation point due to physiological limits on motor unit recruitment consistent with Henneman’s size principle (Henneman et al., 1965). To prevent muscle fatigue, our test protocol involved the participant performing three rounds of gripping at 25%, 50%, and 75% of their maximum voluntary contraction (MVC) for 3 s, each followed by a 3-s rest. The participant completed three trials for each armband and fabric condition. Our data exhibited three distinct EMG signal sets, with amplitudes correlating to the respective gripping force levels under different fabrication conditions as depicted in Figure 3(b).

The results of the textile EMG sensors underscore the significance of selecting an optimal armband size that ensures superior signal quality without sacrificing wearer comfort (Li et al., 2021), as detailed in Table 2.

**Table 2.** Signal-to-noise ratio (SNR_dB_) of EMG signals from textile EMG sensors

Fabrication conditions		SNR (dB)					
		S10	S20	S30	I10	I20	I30
**Strain (%)**	**5**	15.65 ± 0.72	19.24 ± 2.27	19.91 ± 1.42	16.04 ± 2.29	22.05 ± 0.48	17.76 ± 0.99
	**10**	18.84 ± 1.23	21.76 ± 1.99	24.86 ± 1.69	18.51 ± 1.61	23.20 ± 1.25	20.14 ± 0.22
	**15**	-	-	-	19.14 ± 0.56	19.55 ± 1.27	19.69 ± 1.12
	**20**	-	-	-	-	-	16.00 ± 0.83

*Note:* The textile EMG sensors were fabricated based on Table 1. SNR of the conventional wet electrode is 25.89 ± 0.24. All data are reported as mean ± standard deviation.

**Figure 4.**
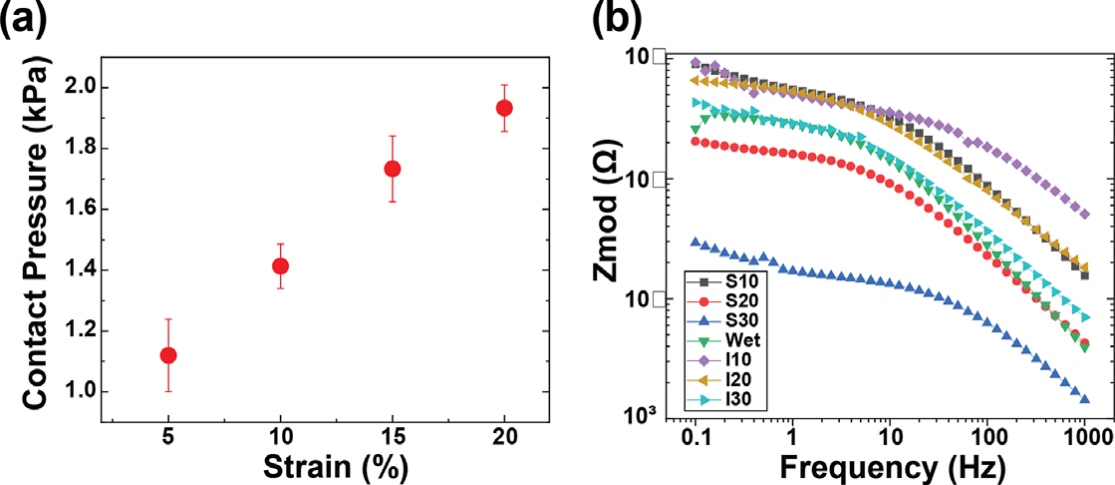
(a) Average contact pressure between skin and electrodes according to the strain level controlled by the velcro strips on the armband, and (b) skin-electrode impedance of textile EMG sensors with various fabrication conditions.

Remarkably, no EMG signals were recorded from screen-printed electrodes beyond a 15% strain. Only inkjet-printed electrodes with a 30 mm diameter reliably captured EMG signals up to a 20% strain. This results from the conductive layer thickness and rigidity limiting the fabric stretchability because a thicker layer is more prone to cracking. (Yokus et al., 2016; Kim et al., 2019) Due to their resilience against deformation during wear, inkjet-printed paths boasted a more extensive strain range for sensing performance compared to their screen-printed counterparts. As illustrated in Figure 3(a) and (b), the screen-printed electrodes at 5% strain outperformed those at 10% strain in terms of SNR_dB_ across all electrode diameters. Figure 4(a) demonstrates that the contact pressure is significant in acquired good biosignals, specifically in the 1–1.5 kPa range. This aligned with the literature on the ideal contact pressure range for optimizing the armband’s functionality and comfort for biopotential measurements (Knowles et al., 2022; Youn et al., 2023; Youn et al., 2023). Screen-printed electrodes with a larger diameter consistently demonstrated a higher SNR_dB_ under strains of 5 and 10%. Conversely, inkjet-printed electrodes indicated an SNR_dB_ plateau or even a slight decline in sensing performance across strains of 5, 10, and 15%. It is worth noting that the skin-electrode impedance plays a pivotal role in capturing high-fidelity EMG signals. Higher skin-electrode impedance invariably translates to a lower SNR_dB_, as the high impedance reduces the signal amplitude channeled to the subsequent amplifier. It is evident in Figure 4(b), where screen-printed samples consistently exhibit a lower impedance and consequently a higher SNR_dB_ than their inkjet-printed counterparts, even when diameters are held constant. This is primarily due to the compositional difference between the inks for screen printing and inkjet printing, which are Ag/AgCl and pure Ag, respectively.

**Figure 5.**
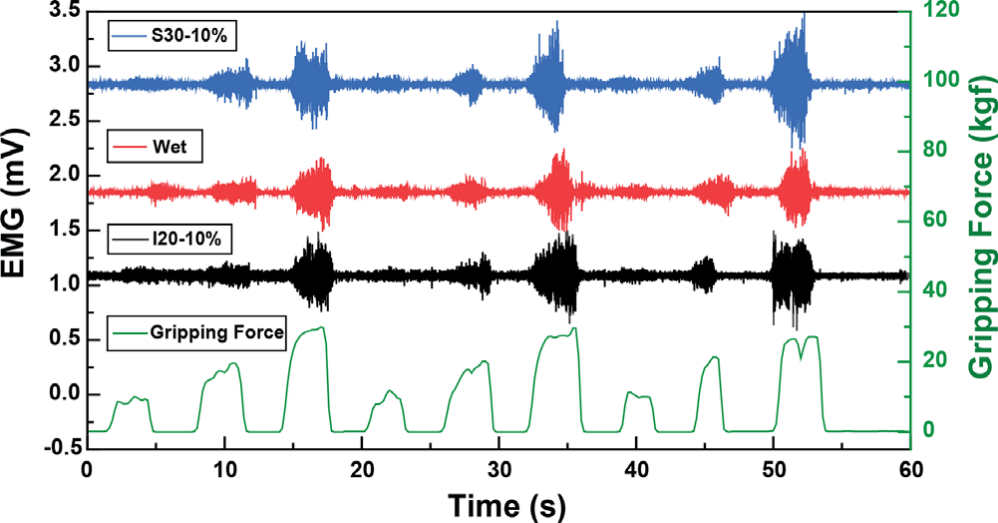
EMG signals under optimal fabrication conditions for inkjet-printed electrodes (I20–10%) and screen-printed electrodes (S30–10%) compared with the traditional wet electrodes according to the gripping force in real-time.

Furthermore, the form factor of electrodes poses a significant influence on the EMG sensing performance. Excessively large electrode diameters can misposition the bipolar electrodes, preventing their proper alignment with the target muscle. The inter-electrode distance must not surpass a quarter of the muscle fiber length, especially in the context of smaller muscles based on the SENIAM recommendations (Stegeman and Hermens, 2007). In light of this, screen-printed electrodes of larger diameters reflected a pronounced decrease in skin-electrode impedance. This observation aligns with the higher SNR_dB_ findings presented in Figure 5 despite the inherent challenges of larger diameters present for EMG sensing. On the other hand, while inkjet-printed electrodes of higher diameters showed a slight decrease in skin-electrode impedance, the electrode form factor was observed to exert a pronounced influence on the SNR_dB_, particularly for diameters exceeding 20 mm.

We evaluated the impact of electrode surface area and strain induced by armband sizing on EMG sensing capabilities. The textile EMG sensors tailored under optimal fabrication conditions that rendered the highest SNR_dB_ (S30–10% and I20–10%) were compared with conventional 20 mm wet Ag/AgCl electrodes. Remarkably, EMG signals from the optimum printed dry electrodes were well-matched to the gripping force in real-time according to the gripping force level. Furthermore, sample S30–10% exhibited an SNR_dB_ of 24.86 ± 1.69 dB, sample I20–10% showed an SNR_dB_ of 23.20 ± 1.25 dB, and the wet electrodes’ SNR_dB_ was 25.89 ± 0.24 dB. These metrics assure the reliability of EMG data acquisition using the printed electrodes. We concluded that our innovative textile EMG sensors hold the potential to replace conventional wet electrodes, which are not reusable and comfortable. Our analysis pinpointed 10% as the optimal strain for both printing methodologies, considering contact pressure and the resistance breakpoint. While the choice of an optimal diameter depends upon skin-electrode impedance, the 20 mm diameter emerges as a frontrunner, especially when material efficiency and design constraints are considered. It is important to note that the observed differences in material performance and the correlation between electrode diameter and EMG SNR require further validation. Future studies would involve larger sample sizes, combined with interpolation and extrapolation methods to rigorously evaluate this relationship.

### 3.1. Use-case scenarios with a wearable telehealth biofeedback system

**Figure 6.**
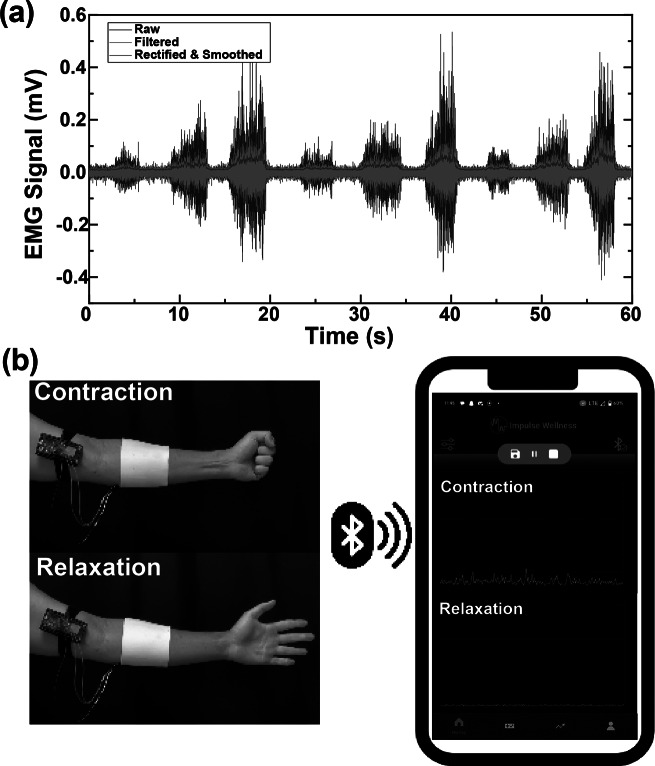
(a) Signal processing steps of EMG for mobile application, including rectification and smoothing. (b) PCB design for the amplifier puck for the textile EMG sensors.

Noise in EMG data that predominantly stems from motion artifacts and common mode interference blurs genuine muscular activity signals. To counteract this, the BEST system applies a second-order Butterworth band-pass with f3dB points of 10 Hz and 250 Hz within the mobile application. Additionally, the mobile application rectified and smoothed the data, as depicted in Figure 6(a). By incorporating our textile EMG sensors with dry electrodes into a mobile TH system, we showcased the feasibility of collecting clinic-quality data in externally valid settings that are often challenging for traditional biosignal technologies, as illustrated in Figure 6(b).

**Figure 7.**
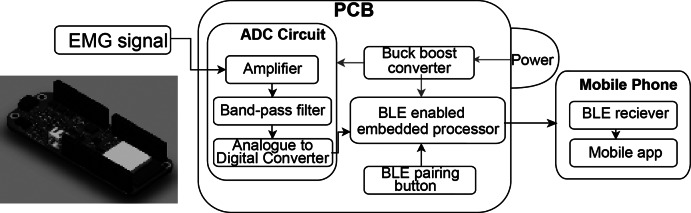
PCB design for the amplifier puck for the textile EMG sensors developed by Impulse Wellness. (3D model was created by Fusion360).

Our textile EMG sensors, paired with the PCB-embedded system and mobile application (Impulse Wellness), ensure reliable data acquisition and contribute added value by being reusable and highly adaptable to telehealth applications as shown in Figure. 7. This system harnesses the potential of our textile-based dry electrodes, broadcasting the EMG data through Bluetooth Low Energy (BLE), and advanced signal processing strategies. Specifically, our system supports remote EMG data monitoring via mobile devices without requiring in-person appointments, thereby demonstrating its practical adaptability in home and clinic settings. Furthermore, the application delivers individualized feedback to users and their healthcare providers. Integrating our textile technology within the wearable TH system serves as a model demonstrating how such advancements can enhance traditional biosignal systems. This integration suggests that other biosensors equipped with textile-based electrodes can reach broader markets more seamlessly, offering greater adoption potential and versatile application in various therapeutic contexts.

Beyond mere data presentation, developing EMG sensors in a wearable and transportable system enables the development of both continuous physiologic monitoring and diagnostics. Such TH systems can apply post-processing methods, incorporating various biomarkers to indicate a wearer’s motor function and potential recovery status. These techniques, like calculating biomarkers such as IMC and muscle ratios, will include the biofeedback algorithm offering a distinct value indicative of the user’s motor function during a task. A lower score implies limited volitional activity or high antagonist co-contraction, as captured by our device. Conversely, a higher value symbolizes improved muscle activation patterns akin to an ideal motor attempt characterized by consistent amplitude targets and minimal antagonist co-contraction. Offering a personalized experience, users can tweak the processing algorithm specifics, tailoring them to specific tasks and individual requirements. This adaptive scoring, complemented by real-time muscle activity visualization on the device, enriches the user experience. With intricate computations operating subtly in the background, the user perceives the application as an engaging game, consistently aiming to elevate their performance score In this context, there is a promising avenue in using garment-embedded physiologic monitoring technology for evaluating fine motor skills, a critical metric in identifying sensory-motor dysfunction associated with neurological disorders such as Stroke, Alzheimer’s, Parkinson’s, epilepsy, and autism such as the Finger Tapping Test (FTT) (Amit et al., 2020).

For example, with our developed EMG sensors connected with the amplifier puck from Impulse Wellness, we targeted the FDS muscle as it is pivotal in evaluating hand movements and advanced hand motions, which are essential mobility metrics on the Motor Assessment Scale (MAS). MAS is a performance-centered scale often used to gauge the degree of impairment and routine motor functions in stroke patients. This intricately assesses hand movements such as radial deviation of the wrist and elbow pronation/supination. Moreover, advanced hand motion assessment on the MAS involves tasks like picking up everyday items like pens, spoons, and jellybeans and activities such as drawing lines or dots with a pen (Carr et al., 1985).

Branching out from the medical fields, potential applications extend to military and VR/AR domains. Our EMG device is designed to revolutionize military health tech by monitoring soldiers for fatigue-related biomarkers, thus preventing musculoskeletal overuse injuries. Additionally, as we venture into the expanding metaverse, we expect multimodal wearable sensors that blend printed electrode arrays with other sensors like strain or pressure sensors (Ju et al., 2021). This innovative system is set to redefine AR/VR interactions by accurately tracking hand motions and rotations, offering users an unparalleled immersion into virtual realms through muscle-signal-driven feedback.

## 4. Conclusion

This work evaluated the effect of the printed dry electrode size and the design of the textile EMG sensors and their efficacy for integration with TH biofeedback technologies. We investigated the effectiveness of our textile EMG sensor’s fabrication conditions, including electrode area and sizing for contact pressure. These sensors, when compared to the traditional 20 mm wet electrodes, showcased reliable EMG collection, with SNR_dB_ values being notably comparable. We further established that the optimal strain for EMG sensing performance is 10% across both printing methods. Given the constraints of skin-electrode impedance, a 20 mm diameter emerged as the most efficient in terms of both material and design. These findings pave the way for the development of highly reliable and reusable dry textile electrodes that alleviate many of the challenges posed by traditional wet electrodes. Moreover, our system is capable of being fully integrated within wireless EMG amplifier systems and has successfully addressed key challenges in EMG readings, such as motion artifacts and common mode interference significantly reduced through effective contact pressure control. The development of a wearable garment system that may be supplemented with transportable electronics and mobile applications such as the BEST system elevates the user experience with regard to biosignal collection and monitoring. This collective system not only offers detailed real-time muscle activity visualization but also provides TH technologies with unrivaled access to vital health-related information and has the potential to redefine the landscape of TH technologies by promoting usability, comfortability, and accessibility of biofeedback technologies. Notably, this study represents a feasibility evaluation conducted in collaboration with a startup company. Additional subjects will be included in future work to further validate our findings. The pin connection can be made more robust by reinforcing it with conductive epoxy and potentially using a heavier fabric that could support the pin well. Future work will optimize the armband design for the general population and include human trial studies to assess the influence of different body types.

## Data Availability

All research data curated and organized by the authors are available upon request and in accordance with data-sharing policies.
